# Extracorporeal membrane oxygenation for the anesthetic management of a patient with a massive intrathoracic goiter causing severe tracheal obstruction with positional symptoms: A case report

**DOI:** 10.1097/MD.0000000000017650

**Published:** 2019-10-18

**Authors:** Young-Il Jeong, In-Gu Jun, Seung-Soo Ha, Hyun-Jung Kwon, Yu-Mi Lee

**Affiliations:** Department of Anesthesiology and Pain Medicine, University of Ulsan College of Medicine, Asan Medical Center, Seoul, Republic of Korea.

**Keywords:** extracorporeal membrane oxygenation, goiter, tracheal obstruction

## Abstract

**Introduction:**

Perioperative anesthetic management in cases of severe airway obstruction with positional symptoms can be associated with difficulties in ventilation or intubation, with a risk of acute respiratory decompensation at every stage of anesthesia.

**Patient concerns:**

Here we describe the anesthetic management of a 67-year-old man with a massive intrathoracic goiter causing severe tracheal obstruction with positional symptoms. The patient presented with progressive dyspnea that was aggravated in the supine position and was scheduled for total thyroidectomy.

**Diagnosis:**

Preoperative computed tomography showed a large goiter extending into the thoracic cavity, with diffuse segmental tracheal narrowing for 6 cm. The diameter at the narrowest portion of the trachea was 4.29 mm.

**Interventions:**

Before the induction of general anesthesia, we applied extracorporeal membrane oxygenation (ECMO) in preparation for potential difficulties in securing the airway during general anesthesia. Subsequently, anesthesia was successfully induced and maintained.

**Outcomes:**

After the surgical procedure, fiberoptic bronchoscopy and chest radiography showed resolution of the tracheal narrowing. ECMO was weaned 2 hours after the surgery, and the patient was extubated on the first postoperative day. He was discharged without any complication.

**Conclusion:**

The findings from this case suggest that the use of ECMO before the induction of general anesthesia is a safe method for maintaining oxygenation in patients with severe tracheal obstruction.

## 1 Introduction

Perioperative anesthetic management in cases of severe airway obstruction with positional symptoms can be associated with difficulties in ventilation or intubation, with a risk of acute respiratory decompensation at every stage of anesthesia. In particular, patients with anterior mediastinal masses are at a higher risk of perioperative complications, which can occur while placing the patient in the supine position, during the induction of anesthesia, at the time of extubation, or even a few days after extubation.^[1]^ Mediastinal tumors do not form a homogenous group. Although many case reports have described the occurrence of decompensation following the induction of anesthesia in patients with mediastinal tumors,^[2–5]^ there are no standard protocols that can guide the perioperative procedures in such cases.

Extracorporeal membrane oxygenation (ECMO) is a life support technique for maintaining both cardiac and respiratory functions through the use of mechanical devices. It is used for the management of acute, severe, reversible respiratory or cardiac failure refractory to conventional management in several tertiary care centers.^[6]^ Here we describe the use of ECMO for the anesthetic management of an elderly patient with a massive intrathoracic goiter causing severe tracheal obstruction with positional symptoms.

## 2 Case report

A 67-year-old man with a massive intrathoracic goiter presented to the Department of Endocrine Surgery with recent onset of wheezing, cough, and dyspnea on exertion and in the supine position. Investigation revealed tracheal compression by the goiter, and total thyroidectomy without neck dissection was scheduled.

**Figure 1:**
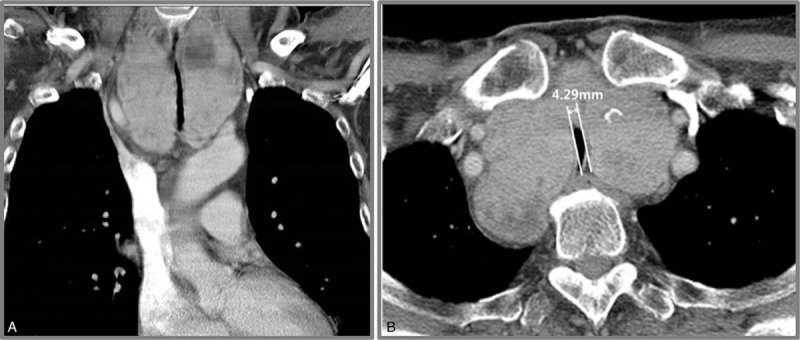
Preoperative CT findings for an elderly patient with a massive intrathoracic goiter causing severe tracheal obstruction with positional symptoms. CT shows a large multinodular goiter extending to the thoracic cavity, with diffuse segmental tracheal narrowing for 6 cm (A). The diameter at the narrowest portion of the trachea is 26 × 4.29 mm (B). CT = computed tomography.

The patient's height and weight were 176 cm and 72 kg, respectively. His medical history included diabetes mellitus, hypertension, and acromegaly with pituitary adenoma. There was no history of allergy. Lung auscultation revealed coarse sounds during both inspiration and expiration. Preoperative laboratory tests returned normal findings, while preoperative computed tomography (CT) demonstrated a massive goiter with multiple low-attenuation nodules extending into the thoracic cavity and causing extrinsic airway compression. Severe tracheal obstruction caused by diffuse segmental slit-like narrowing for 6 cm was observed (Fig. 1A). The lumen diameter at the narrowest portion of the trachea was 26 × 4.29 mm (Fig. 1B). The goiter encapsulated and mildly compressed vessels such as the right brachiocephalic artery and vein and the left common carotid artery without invasion. However, there was no hemodynamic instability. Pulmonary function tests in the upright position showed a forced expiratory volume in 1 second (FEV1) of 1.86 l (53% predicted), forced vital capacity (FVC) of 2.77 l (57% predicted), and FEV1/FVC ratio of 67%. Thus, a mixed obstructive and restrictive pattern was present.

**Figure 2:**
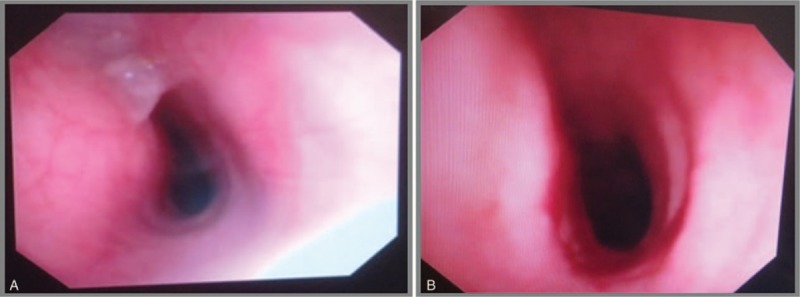
Findings of fiberoptic bronchoscopy for an elderly patient with a massive intrathoracic goiter causing severe tracheal obstruction with positional symptoms. Bronchoscopy reveals near total collapse of the trachea before surgery (A) and resolution of the tracheal narrowing after total thyroidectomy (B).

Preoperative bronchoscopy revealed almost total collapse of the trachea, as expected on the basis of the CT findings (Fig. 2A). The respiratory physicians deemed advancement of a flexible fiberoptic bronchoscope (Model BF-240; distal end diameter: 5.9 mm; Olympus Optical, Tokyo, Japan) impossible because of the risk of tracheal rupture during preoperative bronchoscopy. Considering the possibility of total airway obstruction during the induction of anesthesia, they strongly recommended the use of ECMO before induction. Accordingly, we applied venovenous ECMO before inducing anesthesia.

Premedication was not used. A hemodynamic monitoring system (electrocardiography, noninvasive arterial blood pressure measurement, and pulse oximetry) was set up and train-of-four monitoring initiated. Initial vital signs were as follows: blood pressure (BP) of 178/73 mmHg, regular sinus rhythm with a heart rate (HR) of 58 beat/minute, and peripheral oxygen saturation (SpO_2_) of 99%. The right radial artery was cannulated using a 20-gauge cannula for invasive arterial pressure monitoring, and a 16-gauge peripheral intravenous cannula was inserted in each arm. The patient was preoxygenated with 100% oxygen using a facemask. Dexmedetomidine was infused at 1 mcg/kg over 10 minutes, followed by continuous intravenous infusion at 0.5 mcg/kg/hour. Spontaneous ventilation was maintained. Heparin 4000 U was administered, and a cardiac surgeon who was present in the operating theatre before induction proceeded with percutaneous cannulation of the femoral vessels under local anesthesia. Then, an ECMO circuit (CAPIOX EBS, Terumo Corporation, Tokyo, Japan) primed with plasma solution (500 ml) was applied. The flow rate was 4500 ml/minute, and the activated coagulation time was 319 seconds. Following ECMO initiation, BP, HR, and SpO_2_ were 155/70 mmHg, 55 beat/minute (regular sinus rhythm), and 100%, respectively.

Under continuous intravenous infusion of dexmedetomidine 0.5 mcg/kg/hour, anesthesia was induced by intravenous injection of rocuronium 0.7 mg/kg. The patient was manually ventilated with a tidal volume of 8 to 10 ml/kg, maintaining a respiratory rate of 8 to 12 breaths/minute with 100% oxygen. Subsequently, target-controlled infusion (TCI) of 2% propofol and remifentanil was initiated; the bispectral index (BIS; BIS A-1050 Monitor; Aspect Medical Systems, Newton, MA) was maintained between 30 and 40.

**Figure 3:**
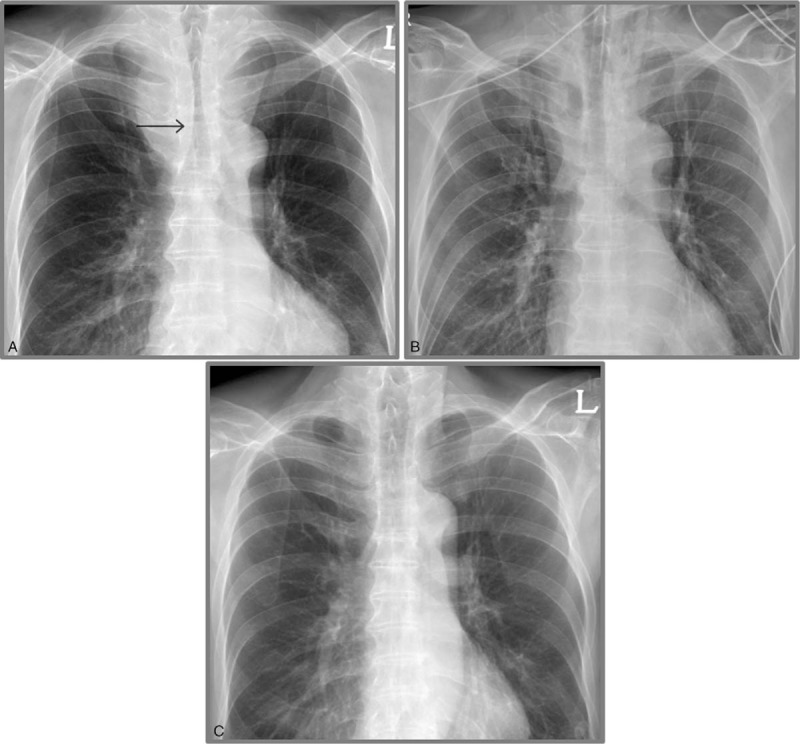
Perioperative chest radiographs for an elderly patient with a massive intrathoracic goiter causing severe tracheal obstruction with positional symptoms. The patient received extracorporeal membrane oxygenation before anesthesia induction. The tip of the endotracheal tube is placed at a depth of 28 cm (arrow line) beyond the narrowest portion of the trachea (A). After the surgical procedure, the endotracheal tube is exchanged with a silastic cuffed tube, which is fixed at a depth of 23 cm (B). A postoperative chest radiograph shows restoration of the tracheal lumen after extubation (C).

Tracheal intubation with a No. 6 oral tube (Mallinckrodt^TM^ oral endotracheal tube with a TaperGuard^TM^ Cuff; Covidien, Dublin, Ireland) was performed under bronchoscopic guidance (model XBF-3B40Y1; outer diameter, 3.5 mm; Olympus Optical, Tokyo, Japan). The endotracheal tube was advanced beyond the narrowest portion of the trachea, and the 28-cm mark was initially fixed at the right oral commissure (Fig. 3A). Then, dexmedetomidine infusion was terminated and the patient was mechanically ventilated under the following parameters: tidal volume, 8 to 10 ml/kg; respiratory rate, 5 to 10 breaths/minute; end tidal carbon dioxide partial pressure, 20 to 35 mmHg; peak inspiratory pressure, 10 to 20 cmH_2_O; and 50% oxygen (medical air). There was no difficulty in delivering positive-pressure ventilation. Anesthesia was maintained by TCI of propofol 2 mcg/ml (effect–site concentration) plus remifentanil 2 to 5 ng/ml (effect–site concentration), with a BIS of 30 to 40.

For the surgical procedure, the neck was propped against a pillow and kept extended in the supine position. Following sterilization and draping of the surgical field, a low-collar incision was placed and the muscles and vessels around the thyroid gland were dissected and ligated, respectively. The thyroid gland was successfully resected, and the surgical site was irrigated by warm saline. A Jackson–Pratt drain was inserted. Finally, the wound was closed layer by layer. The operative course was uneventful, and the perioperative vital signs are detailed in Table 1.

**Table 1:**
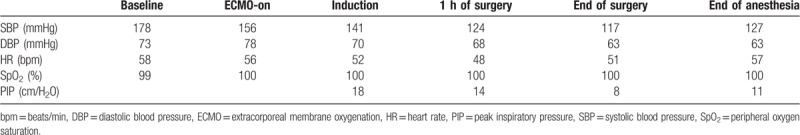
Perioperative vital signs and PIP values for an elderly patient who received ECMO before anesthesia induction during surgery for a massive intrathoracic goiter causing severe tracheal obstruction with positional symptoms.

Resolution of the tracheal narrowing was confirmed by fiberoptic bronchoscopy performed after the surgery (Fig. 2B). Therefore, the endotracheal tube was exchanged with a No. 7 silastic cuffed tube, with the 23-cm mark fixed at the right oral commissure (Fig. 3B). The patient was transferred to the intensive care unit. ECMO was weaned 2 hours after the surgery. The femoral cannulae were also removed. The total pump duration was 4 hours and 55 minutes. Extubation was uneventfully performed on the first postoperative day. A postoperative chest radiograph showed restoration of the tracheal lumen (Fig. 3C). The patient was transferred to the general ward the following day and discharged without any complications on the fifth postoperative day. The patient has provided informed consent for publication of the case.

## 3 Discussion

General anesthesia in patients with a large mediastinal mass causing airway and cardiovascular compression is always challenging and critical. Since Bitter described a case of acute airway obstruction associated with the induction of general anesthesia in a patient with mediastinal Hodgkin's disease in 1975, a number of cases alerting anesthesiologists about the risk of general anesthesia in patients with mediastinal masses has been reported.^[7]^

Acute respiratory decompensation may be precipitated by positional changes, the loss of muscle and diaphragm tone, and alterations in the lung compliance and chest wall structure not only during the induction of anesthesia but also during and after the surgical procedure.^[8]^

Thorough preoperative assessments involving history taking, physical examinations, and imaging studies are considered very important for the identification of patients at risk of airway complications. Patients with a high risk of perioperative complications can be identified by the presence of cardiorespiratory signs and symptoms at presentation, CT studies (tracheal compression, >50% or pericardial effusion), and mixed abnormalities (i.e., combined obstructive and restrictive patterns) in pulmonary function tests.^[1,9]^ Chest CT can demonstrate tracheal and bronchial compression and permits accurate measurements of the airway diameters. In addition, it helps in determining the precise level and extent of compression of the tracheobronchial tree.^[4]^ Flow-volume loops are commonly ordered as part of preoperative assessments for patients with an anterior mediastinal mass. Specifically, an increased midexpiratory plateau during a transition from the upright to the supine position is considered pathognomonic of variable intrathoracic airway obstruction and a predictor of patients at risk of airway collapse during the induction of anesthesia.^[9,10]^

To prevent complications during anesthesia, awake flexible fiberoptic intubation with the maintenance of spontaneous ventilation before induction can be considered if an endotracheal tube can be advanced to the distal noncompressed airway beyond the obstruction.^[9]^ Changing the patient position to minimize symptomatic airway compression or immediate ventilation with rigid bronchoscopy distal to the obstruction by an experienced bronchoscopist are treatment options in the event of life-threatening airway collapse during surgery.

Despite the maintenance of spontaneous ventilation, however, extreme difficulty in maintaining the airway patency and a severe decrease in oxygen saturation have been reported. Thus, maintenance of spontaneous ventilation cannot assure airway patency during anesthesia. In a previous study involving such cases, airway rescue and hemodynamic decompensation was achieved in some cases by emergent femorofemoral bypass by surgeons kept on “standby” in the operation room. In a few cases, however, fatal neurologic deficits were observed.^[11]^

Airway compression due to a mediastinal mass may occur immediately after the induction of general anesthesia. Changing the patient's position to take the tumor weight off the trachea or main bronchus may improve oxygenation. However, this did not happen in our patient, probably because of the size of the goiter. The use of ECMO in patients with refractory hypoxemia and hypotension is another strategy that can be considered.^[3,5]^ ECMO is a life support technique for maintaining both cardiac and respiratory functions by using mechanical devices when the native systems fail.^[12]^ It is of 2 types: venoarterial ECMO, which has the potential to provide complete respiratory and hemodynamic support, and venovenous ECMO, which supports only the respiratory system and allows gas exchange outside the body.^[13]^ In high-risk patients classified as “unsafe,” decompensation after anesthesia induction should be expected and the option of connection to an extracorporeal circulation must always be provided. The femoral vessels should be cannulated under local anesthesia before surgery.^[11]^ Recent studies recommend the establishment of an alternative method (ECMO or cardiopulmonary bypass) for oxygenation before the induction of anesthesia in patients classified as “unsafe” and “uncertain,” thus opposing the “standby” system for cases of emergency. In particular, patients with severe positional symptoms due to airway or cardiovascular compression cannot be induced safely. In case of cardiopulmonary decompensation, at least 5 to 10 minutes are required for cannulation and establishment of adequate circulation and oxygenation, even if a primed pump and prepared team are present. Such delays can lead to adverse neurological sequelae.^[11]^

In the present case, ECMO was initiated via femorofemoral bypass prior to the induction of anesthesia in order to avoid fatal perioperative complications associated with potential airway collapse during anesthesia. Establishment of ECMO before induction was advised by an experienced bronchoscopist who had performed preoperative bronchoscopic examinations and did not attempt to bypass the scope through the severely constricted tracheal lumen. Initially, we considered awake fiberoptic intubation and the maintenance of spontaneous ventilation; however, we did not implement the same because we could not ensure airway patency throughout the surgical procedure. Moreover, there was a concern that prolonged forced advancement of an endotracheal tube with a diameter that does now allow access to the trachea beyond the obstructive lesion without injury would cause complications such as tracheal mucosal laceration, hemorrhage, edema, pressure-induced ischemic injury, and stricture formation. Accordingly, we decided to establish ECMO before induction and used a short endotracheal tube with a smaller diameter in order to access the distal trachea with minimal airway trauma. Although studies on the use of extracorporeal circulation before the induction of anesthesia in order to prevent airway and hemodynamic complications are limited to a few case reports, establishment of a femorofemoral bypass under local analgesia before anesthesia induction has been safely performed as an elective (not rescue) procedure in adult patients.^[6]^

ECMO does not come without potential risks, including vascular injury and hemorrhage at the puncture site, bleeding due to heparinization, thromboembolism, lipid deposition on the oxygenator membrane due to propofol infusion, sequestration of other drugs in the circuit, and mechanical failure resulting in hypoxia. Therefore, the risks and benefits should be weighed before a decision is made. Moreover, consideration about cannulation and priming of the ECMO circuit (not in a “standby” manner) or further initiation of ECMO before induction may be another issue. Nevertheless, because ECMO and cardiopulmonary bypass are now considered safe and acceptable modalities for daily clinical use, they can be considered as oxygenation techniques in select cases.^[6,11]^

In conclusion, our findings suggest that the induction of general anesthesia is risky in patients with severe tracheal obstruction causing positional symptoms, even with the maintenance of spontaneous ventilation. The use of ECMO before the induction of general anesthesia may be a safe and effective strategy for maintaining oxygenation in such patients. Safe and calm management is always preferable over hurried decisions in the event of acute cardiopulmonary compromise.

## Author contributions

**Conceptualization:** Yu-Mi Lee.

**Data curation:** Hyun-Jung Kwon.

**Investigation:** Yu-Mi Lee.

**Methodology:** In-Gu Jun.

**Project administration:** Yu-Mi Lee.

**Software:** Young-Il Jeong, Seung-Soo Ha.

**Supervision:** Yu-Mi Lee.

**Validation:** Young-Il Jeong.

**Writing – original draft:** Yu-Mi Lee.

**Writing – review & editing:** Yu-Mi Lee.
